# Predicting Oral Cancer From Precursor Lesions: The Case for a Standardized Framework of Analysis to Improve Prediction Modeling

**DOI:** 10.1002/hed.70465

**Published:** 2026-09-24

**Authors:** Michael E. Troka, James C. Gates

**Affiliations:** ^1^ Division of Oral and Maxillofacial Surgery Mayo Clinic Rochester Minnesota USA; ^2^ Department of Oral and Maxillofacial Surgery University of Pennsylvania Philadelphia Pennsylvania USA

## Abstract

Oral Cancer often occurs from the transformation of precursor lesions, and this offers an opportunity for early detection. Current methods to assess risk of precursor lesion progression to oral cancer incompletely predict risk. A multimodal framework that leverages machine learning is needed to improve prediction. In this manuscript, we propose more standardization in the study of OPMD across sites and propose a framework aligned to the principles of the NCI's Human Tissue Atlas Network (HTAN). We explore whole exome sequencing for genomic risk, methylation array for environmental genetic regulation, RNA sequencing for transcriptional control of cellular programs with spatial transcriptomics to fulfill the spatial requirements of HTAN. Moreover, we propose a machine learning (ML) analysis whereby a learned image‐molecular association is created by integrating H&E histology and sequencing results. Once these associations are learned, a “Teacher‐Student” model can be created whereby the Student model can be deployed to infer the “molecular associations” from H&E and patient covariates alone. Such a framework integrating multiomic data across multiple different biologic processes and time points can help to better understand and potentially even predict the biologic events that underlie oral cancer initiation.

## 1 Background: The Clinical Problem

Oral precancerous lesions, or oral potentially malignant disorders (OPMDs) as they are called by the WHO, represent a significant concern in clinical oncology due to their potential to develop into oral squamous cell carcinoma (OSCC) [1]. OSCC is a cancer with about 60%–70% 5‐year mortality and significant treatment side effects, impacting speech, eating, appearance, with the potential to cause lasting dysfunction and visible stigma [2]. The pooled incidence of malignant transformation for dysplastic OPMDs approaches 15%, creating a clinical dilemma in determining which will progress to cancer and which precancerous lesions do not [3, 4, 5]. Current clinical management is controversial, and the inability to prognosticate may lead to overtreatment while conservative surveillance risks delayed diagnosis [6, 7]. Moreover, treatments for oral cancer are most successful when the disease is detected early, providing the impetus for early detection strategies. However, no validated screening test or early detection method exists to identify which OPMDs require intervention, as exists in other cancers such as breast, prostate, colorectal, or lung cancers [8, 9, 10].

The National Cancer Institute's (NCI) Human Tumor Atlas Network (HTAN) has formalized that to prevent cancer, we must characterize how lesions evolve: from normal tissue to precancer to invasive disease. To do so, they advocate integrating molecular, cellular, morphologic, and spatial information across time and clinical outcomes (Table 1) [11]. In this framework, data are collected to link a lesion's biology to its outcome (whether it remains stable, regresses, or progresses) with sufficiently standardized biospecimen handling, data capture, and imaging annotation to support reproducible, cross‐site computational modeling. This organization is critical for improving our understanding of how cancers develop. This is particularly important in oral cancer, as precursor lesions often transform from a complex, heterogenous interaction between genetic events, epigenetic state, transcriptional programs, and the local microenvironment, which precede malignant transformation. This provides a potential window of opportunity for early molecular detection across these biologic layers.

**TABLE 1:** NCI Human Tumor Atlas Network (HTAN) guidelines for precancer Atlas construction.

Domain	Component	Requirements/specifications	Scientific rationale
I. Biospecimen unit	Longitudinal sampling	Normal → precancer → invasive cancer trajectory Matched timepoint collection when feasible Minimum: Baseline and transformation endpoint	Captures evolutionary trajectory of lesion development; enables identification of early molecular changes preceding transformation
	Outcome annotation	Progressor vs. nonprogressor classification Time to progression/regression documented Clinical outcome follow‐up (minimum 2 years)	Distinguishes lesions by clinical fate; essential for biomarker discovery targeting progression prediction
	Matched normal tissue	Germline DNA (blood/saliva preferred) Adjacent histologically normal epithelium Distant normal mucosa when available	Required for accurate somatic variant calling; establishes patient‐specific baseline for molecular comparisons
II. Characterization unit	Single‐cell profiling	scRNA‐seq or snRNA‐seq (≥ 5000 cells/sample) Cell viability > 80% for dissociation protocols CITE‐seq for surface protein co‐profiling (optional)	Resolves cellular heterogeneity; identifies rare cell populations and transitional states driving malignant transformation
	Spatial profiling	Spatial transcriptomics (Visium, Xenium, GeoMx) Multiplex imaging (CODEX, CyCIF, IMC) Co‐registration with H&E histology required	Maps tumor microenvironment architecture; preserves spatial relationships between cell types and tissue structures
	Bulk molecular profiling	Whole exome sequencing (≥ 100× coverage) DNA methylation arrays (EPIC/850 K or bisulfite‐seq) Bulk RNA‐seq (≥ 30 M reads/sample)	Provides scaffold for single‐cell data integration; enables mutation and methylation profiling at scale
	Digital pathology	H&E whole slide imaging (40× magnification) MITI‐compliant metadata annotation Pathologist‐verified dysplasia grading	Enables computational pathology pipelines; provides morphological ground truth for molecular correlations
III. Data processing unit (HTAN levels)	Level 1: Raw data	FASTQ files (sequencing) Unaligned BAM files Raw TIFF images (imaging)	Archival storage for reproducibility; enables re‐analysis with updated pipelines
	Level 2: Aligned/processed	Aligned BAM files (GRCh38 reference) Stitched OME‐TIFF images Quality‐filtered reads	Standardized preprocessing; input format for downstream analytical pipelines
	Level 3: Derived molecular	Gene expression matrices (counts) Variant call files (VCF) Cell segmentation masks Methylation beta values	Standard input for analysis tools (Seurat, Scanpy); enables cross‐study comparisons
	Level 4: Integrated/summary	Cell type annotations Dimensionality reduction (UMAP/t‐SNE) Integrated multi‐modal objects Predictive model outputs	Final analytical outputs; atlas visualization and machine learning model deployment
IV. Metadata standards	Clinical metadata	Demographics (age, sex, ancestry) Risk factors (tobacco, alcohol, HPV status) TNM staging, dysplasia grade Treatment history and outcomes	Enables clinical correlation studies; supports prognostic model development
	Technical metadata (MITI)	Ischemia time, fixation protocol Antibody clones, conjugation chemistry Sequencing platform, library preparation Batch identifiers for QC	Ensures reproducibility; enables batch effect correction and quality assessment

Abbreviations: CITE‐seq, cellular indexing of transcriptomes and epitopes by sequencing; CODEX, cO‐detection by indEXing; CyCIF, cyclic immunofluorescence; H&E, hematoxylin and eosin; HPV, human papillomavirus; HTAN, Human Tumor Atlas Network; IMC, imaging mass cytometry; MITI, minimum information about tissue imaging; NCI, National Cancer Institute; scRNA‐seq, single‐cell RNA sequencing; snRNA‐seq, single‐nucleus RNA sequencing.

Technological advancements and decreased costs now afford opportunities for such comprehensive molecular characterization that were previously impractical (Figure 1). Rather than developing another unimodal predictor, we propose a multiomic framework. The backbone of HTAN Precancer Atlas (PCA) Centers typically involves expertise in RNA sequencing and spatial transcriptomics. This provides the link between which genes are active and where in the epithelial‐stromal environment they are being transcribed. Digital histopathology also satisfies the morphologic HTAN requirements. In addition, whole exome sequencing and methylation analysis can be considered to further bolster the molecular data requirements and can provide predisposition, somatic mutation, and environmental contexts to the patient and their lesion [11]. This approach rests on the hypothesis that progression risk is encoded across multiple biological layers and that predictive accuracy requires their joint interrogation. By capturing multimodal data combined with clearly defined clinical endpoints, we aim to identify convergent signatures no single modality could reveal.

**FIGURE 1:**
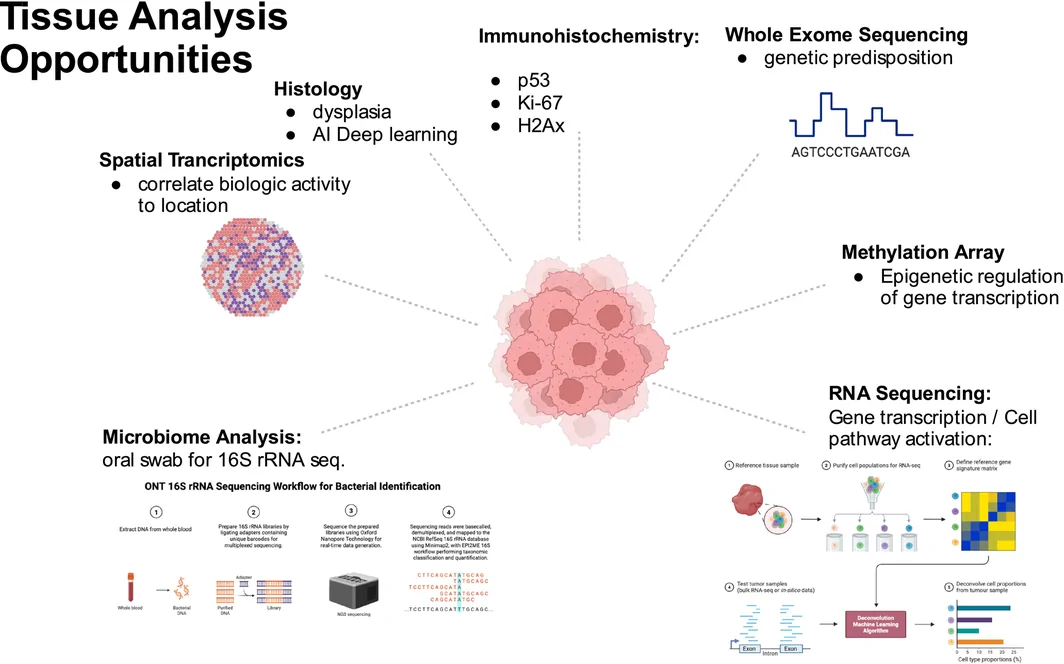
Diagnostic tests for multiomic investigation of biologic events and predispositions that underlie OPMD progression and oral cancer initiation. [Color figure can be viewed at wileyonlinelibrary.com]

The primary aim of such a multiomic project would be to improve risk stratification of OPMD over current methods such as histology or other proprietary or investigational tools. This could provide the basis for multi‐institutional study and development of a predictive algorithm. However, clinical deployment of multiomic profiling faces barriers of cost, turnaround time, and infrastructure. These are significant impediments to the realization of improved prediction and likely why histology remains the current standard. For this reason, as a secondary aim, we propose a Teacher‐Student deep learning architecture, where molecular characterization informs model training which can infer these complex characteristics from routine H&E histology. This would then allow for potential widespread deployment in clinical practice. If successful, this would enable molecular risk stratification based on learned image‐molecular associations. A point of care test could then be developed to expand accessibility and decrease cost from the expensive sequencing used to generate this image‐molecular association algorithm. The following sections detail the study design considerations, specimen requirements, and modality‐specific protocols necessary to execute this framework.

### 1.1 Study Design Considerations for Multiomic OPMD Characterization

Constructing a predictive framework for OPMD progression requires intentional study design. We must align specimen collection, outcome annotation, and data processing with the goal of distinguishing stable from progressing lesions. The NCI HTAN has formalized guidelines for pre‐cancer atlas construction that systematically address these requirements (Table 1). This supports the organization and standardization of studies to ensure that the resulting data are structured to support reproducible computational modeling and cross‐site validation.

The first design consideration is longitudinal sampling. Premalignant lesions exist along an evolutionary trajectory whose endpoint is uncertain at the time of initial diagnosis. Atlas‐grade OPMD characterization therefore requires specimens that span the transition from normal mucosa through dysplasia to invasive carcinoma. Matched normal tissue, ideally germline DNA from blood with histologically normal adjacent epithelium, is essential for accurate somatic variant identification and for establishing patient‐specific molecular baselines. When longitudinal biopsies are available, they enable within‐patient modeling of molecular change over time, providing a more direct view of progression dynamics than cross‐sectional comparisons alone.

Equally critical is outcome annotation. The central question in OPMD prognostics is not what molecular alterations a lesion harbors, but whether those alterations predict clinical outcome. This requires prospective follow‐up with documented endpoints: time to malignant transformation, time to regression or clinical resolution, or time to intervention triggered by high‐grade dysplasia. A minimum follow‐up period of two to 3 years is generally necessary to capture a sufficient number of transformation events for statistical modeling based on power calculations using known transformation rates. The average from diagnosis to meet a transformation depends on the severity of the lesion where those with high‐grade dysplasia are likely to transform sooner than those with lower‐grade or hyperplastic lesions [12]. The largest cohorts all seem to converge on an average time frame of 4–5 years to transformation, including Meci et al.'s recent retrospective analysis of 59 204 patients through the TriNetX database [13, 14, 15]. However, some patients will convert to malignancy earlier, within 2 years, and others will progress to high grade from low grade, and this information is helpful for modeling as well. For these reasons, a 2–3‐year study window will likely provide the minimum data to develop predictive modeling, though certainly longer follow‐up out to 4–5 years improves the reliability of nonprogressor classification when able.

Specimen handling constraints must also be addressed at the design stage. This is because they directly affect which assays are feasible and how data quality varies across the cohort. Fresh tissue processed immediately, snap frozen or preserved in RNAlater, supports high‐quality nucleic acid extraction for whole exome sequencing (WES), methylation arrays, and RNA sequencing, and is required for single‐cell or single‐nucleus profiling. Strict protocols to minimize cold‐ischemia time, the interval between resection and preservation, are essential. In addition, annotation of the exact cold‐ischemia time should be completed. Transcriptional profiles are highly labile and can be distorted by ex vivo stress responses within minutes. FFPE tissue, while common in clinical practice, contains fragmented and chemically modified nucleic acids that limit assay selection and require FFPE‐compatible library strategies. Study design must consider this to ensure valid data can be derived and specimen preservation and processing should be standardized across studies.

Data processing standards ensure that molecular measurements are comparable across samples, sites, and studies. HTAN defines a four‐level data hierarchy (Table 1): Level 1 (raw data such as FASTQ files and unprocessed images), Level 2 (aligned and preprocessed data), Level 3 (derived molecular features such as gene expression matrices, variant calls, and methylation beta values), and Level 4 (integrated summary outputs including cell type annotations, dimensionality reductions, and predictive model scores) [16]. For computational modeling, Level 3 data represent the standard analytical input, while Level 4 outputs are what the model produces [11]. Adopting this hierarchy from the outset facilitates data sharing, reproducibility, and eventual integration with public atlas resources.

Finally, comprehensive covariate and metadata capture is crucial for both biological interpretation and technical quality control. Clinical covariates such as demographics, lesion site, tobacco/alcohol/betel nut exposure, previous oncologic history, size and focal nature of the lesion, comorbidities, and histologic grade must be recorded systematically because they represent confounders, effect modifiers, or independent predictors that a model must account for. Technical metadata—including RNA integrity metrics, fixation duration, library preparation batch, sequencing depth, and array processing batch—must also be documented because technical variation can masquerade as biological signal if not explicitly modeled. The Minimum Information about Tissue Imaging (MITI) guidelines provide analogous standards for histologic and spatial imaging data, specifying metadata requirements similar to those from HTAN [17].

With these design principles established, the following sections detail a linear approach investigating the first half of the central dogma, which is the flow of genetic information. It is proposed as a biologically rationale, multiomic OPMD characterization framework. Admittedly, it does not include proteomics, metabolomics, microbiome consideration, and other aspects of tumor biology. Moreover, there are many other ways to satisfy the morphologic, cellular, molecular, and spatial requirements for HTAN not listed here, and those proposed in this manuscript are the author's proposed implementation. The framework is not intended to be so rigid as to prohibit other iterations and modifications; rather, it should serve as standardized, HTAN aligned research strategies for this disease. Whole‐exome sequencing can be utilized to detect genetic predisposition risk, DNA methylation arrays for epigenetic and environmental risks, RNA sequencing to determine the cellular pathways that are actively being transcribed, and spatial profiling for the location of transcriptional activity found in the microenvironmental context (Figure 1). Each modality presented contributes complementary information, and their integration enables machine learning approaches that can leverage the full dimensionality of the data while remaining anchored to biologically interpretable features and clinically actionable endpoints.

### 1.2 Genomic Profiling: Whole Exome Sequencing

Genomic profiling addresses the most upstream layer of progression biology: the DNA level alterations that shape a lesion's malignant potential. Genetic changes in OPMD are believed to precede and modulate immunologic changes that facilitate oncogenesis [18]. More specifically, inflammatory changes are believed to cause genomic instability, and these mutational changes can lead to immunosuppressive environments that in part permit epithelial cell proliferation. The genetic basis for oncogenesis of lesions to head and neck cancer was originally described by Califano et al. and confirmed to occur retrospectively and then prospectively in OPMD cohorts by Rosin and Zhang et al. This was then further validated in the EPOC trial in 2016 [19, 20, 21, 22]. Our knowledge of the effects of these driver mutations on the lesion microenvironment has been more recently expanded. For example, p53 mutations can lead to “gain‐of‐function” (GOF), pro‐inflammatory, proliferative environments [23], but in others, they potentiate immune suppression. Rather than just looking at whether p53 is mutated or has lost an allele, we appreciate a much more dynamic way in which mutation, loss of heterozygosity, and copy number alterations can occur in different ways to produce distinctly different phenotypic expression in premalignant and malignant lesions [24].

Whole‐exome sequencing (WES) extends this strategy by interrogating coding regions across ~20 000 genes, enabling a more comprehensive view of driver mutations, mutational burden, copy‐number change, and LOH‐like events. Practically, WES is most interpretable when the lesion is paired with a matched normal comparator so true somatic alterations can be distinguished from germline variation. Blood is typically the cleanest source for germline DNA. For the lesion, fresh‐frozen or RNAlater‐preserved tissue generally yields the highest‐quality DNA, but WES can be performed on FFPE tissue when needed, with the caveat that fixation‐related damage and fragmentation can increase artifacts and reduce uniform coverage.

From an analytic standpoint, WES supports several feature classes that map directly onto progression modeling: presence of high‐risk alterations in key pathways, quantitative measures of mutational and copy‐number burden, and metrics of clonal architecture that reflect early evolutionary diversification. Importantly, WES has already been deployed in OPL chemo/immunoprevention trial settings, where higher genomic alteration burden tracked with decreased treatment response and increased progression risk, and where specific genomic events (including chromosome 9p alterations) aligned with higher malignant transformation rates [2, 25, 26]. These data support using WES not only to catalogue mutations but to generate quantitatively modellable genomic risk features in prospective cohorts.

Exome‐scale does carry ethical and logistical implications as it can uncover incidental germline predispositions unrelated to the oral lesion; patients should be counseled and consented accordingly, ideally with access to genetic counseling. Even with these considerations, WES offers a uniquely mechanistic anchor for risk stratification and hypothesis generation, and it is most informative when interpreted as one layer of a convergent progression phenotype rather than as a standalone predictor.

### 1.3 Epigenomic Profiling: DNA Methylation

In OPMDs, altered DNA methylation status is thought to be the result of altered inflammatory and oxidative stress states and may be shaped by chronic exposures such as tobacco, alcohol, friction, trauma and betel nut, supporting the concept that methylation can encode cumulative exposure history and environmental stress [27, 28]. In this framework, we promote examination of changes in methylation pattern, as these changes can be detected before malignant transformation with commercially available tests. Hypermethylation causes suppression of gene transcription, and hypomethylation activates other genes. In OPMD and H&N cancer, hypermethylation has been found to occur at sites of tumor suppressor genes such as CDKN2A [24]. Such suppression of transcription of these important genes has been found to confer a “cold” immune suppressive microenvironment which prevents immune surveillance and destruction of mutated epithelial cells [29].

The combination of such genetic and epigenetic epithelial proliferation in unchecked, permissive immune environments is believed to be the underpinnings of cancer initiation. Although the manifestation of dysfunctional genetic and epigenetic states hasn't been tied to outcomes in OPMD, a more aggressive phenotype or biology is thought to occur in coexisting p53 GOF and CDKN2A hypermethylation [30].

Herein lies the importance of considering both the genetic landscape (mutations, LOH, CNA) as well as methylation states of genes which contribute to oncogenesis in similar, overlapping, or separate manners driving lesions toward invasion.

DNA methylation arrays are well suited for clinical risk stratification in OPMDs because they can be performed directly on extracted DNA [27]. In addition, arrays such as the Infinium MethylationEPIC v2.0 provide broad coverage (~930 000 CpG sites) across functionally annotated regions, enabling quantification of both locus‐specific regulatory changes and broader epigenetic state shifts from a single assay. For modeling, methylation yields feature classes that correlate with progression biology, including differentially methylated positions/regions linked to dysregulated regulatory elements, global hypomethylation metrics as proxies for epigenetic instability, exposure‐ and drift‐associated signatures, and intra‐lesion methylation variability that may reflect epigenetic heterogeneity and clonal diversification. These signals can distinguish lesions that appear similar histologically and mutationally [31].

Thus, genetic and epigenetic profiling should be viewed as complementary rather than redundant. WES identifies sequence and clonal alterations that define genetic risk, while DNA methylation captures regulatory‐state information and field‐level effects that can reflect whether genetic potential is functionally realized. In a stacked framework, integrating WES with methylation—along with transcriptomic and spatial data—allows machine learning to combine discrete genetic events (drivers, copy‐number change, mutational burden) with state variables (regulatory rewiring, field cancerization, and environmental changes that create epigenetic instability) to better distinguish stable from progressing OPMDs, while preserving biologic interpretability of model outputs.

### 1.4 Transcriptomic Profiling: RNA Sequencing

While WES and DNA methylation capture mutational status and a major layer of DNA regulatory accessibility, RNA represents the resultant layer of the central dogma: it is the molecular representation of what the cell is actively doing at a given time. The mRNA present in a sample reflects active transcriptional programs and therefore correlates with cell identity, lineage state, immune activation, differentiation, stress responses, and malignant phenotypes such as proliferation and EMT [32]. Conceptually, WES captures DNA mutations that can alter transcriptional potential, DNA methylation assays reflect whether genomic regions are placed into permissive versus restrictive chromatin contexts, and RNA sequencing reports the downstream consequence—what transcripts are actually produced and at what relative abundance. Stacking these layers provides a more mechanistic view of OPMD progression than any single modality alone.

Significant advancements in our knowledge of transcriptional changes as oral lesions progress from pre‐cancer to cancer have been elucidated. One of the most well‐designed examples was a transcriptional examination of paired tissue samples of normal/adjacent, dysplastic and carcinomatous tissue in nine patients by Sun et al. Using the adjacent tissue as a control, they found enrichment of eight genes/transcription factors as measured by single cell RNA sequencing as lesions progressed to dysplasia and then cancer. These same eight genes were also enriched in the TCGA H&N data set, providing further validation. Moreover, they corresponded to genes and transcription factors implicated in the same processes detected by alteration in the genomic and epigenomic layer: epithelial proliferation, immune suppression and activation of cancer associated fibroblasts [32]. A similar study was conducted on paired OPMD (*n* = 9) and SCCa (*n* = 11). They found shared expression of genes between the two groups which were further enriched in the cancer specimens and these transcription factors correlated to pathways involved in basement membrane disruption, cell migration and metastasis. They developed a five gene set that validates histologic diagnosis (OPMD vs. cancer) that they believe may aid in predicting cancer from OPMD and are awaiting further validation [33]. Similar transcriptional panels have been developed by other groups to predict OPMD progression to cancer [34, 35], though none have survived external validation at the population level, and none include genomic, methylation, proteomic or other multiomic strategies.

Furthermore, single cell transcriptional studies shed light on which cell populations become more abundant as OPMD lesions progress to cancer. For example, regulatory T cells and polarized M2 macrophages are increased in abundance and are believed to lead to exhaustion of T cells that would normally provide immune surveillance, in addition to evidence of impairment of dendritic and natural killer cells and other mechanisms of immune suppression creating a permissive immune environment ripe for epithelial cell proliferation [36, 37, 38].

As we have alluded to, specimen constraints in OPMD trials are central to sequencing platform selection. Fresh tissue processed immediately, or preserved in RNAlater or snap‐frozen, best supports high‐quality bulk RNA‐seq and enables single‐cell profiling in selected cases. In contrast, FFPE tissue contains fragmented and chemically modified RNA. This does not prevent transcriptomic profiling, but it does mean method selection for FFPE‐compatible library strategies is crucial, as well as emphasis on RNA quality metrics and batch‐aware computational processing.

From a modeling standpoint, RNA provides features that are intimately related to cellular phenotype: gene‐level expression, pathway activity, and signatures reflecting inflammation, differentiation, and stress [32]. Computational deconvolution adds further value by allowing researchers to digitally infer the composition of the immune and stromal microenvironment from a single bulk assay. We advocate for a tiered collection strategy that maximizes both scalability and biological resolution: bulk RNA‐seq for the full cohort when fresh‐frozen or RNAlater‐preserved tissue is feasible; an FFPE‐compatible transcriptomic workflow for archival and FFPE‐only specimens; and higher‐resolution single‐cell or single‐nucleus profiling in a strategically selected subset to define progression‐associated cell states and improve interpretability of bulk‐derived transcriptomes.

### 1.5 Spatial Profiling: Spatial Transcriptomics

While decoding the cellular pathways, programs and populations present in OPMD are revealed by RNA sequencing, their relations to another and their environment are incomplete without identifying their spatial positions. Spatial transcriptomics (ST) gives context to gene expression, localizing these findings to specific areas of the tissue (i.e., specific layers of epithelium or stroma). In addition, ST can provide a temporal relationship of transcriptional changes along the course of disease progression. This makes it a key component of the framework and complementary to RNA sequencing.

Sun et al. utilize ST to complement RNA sequencing (RNA seq) to demonstrate the spatio‐temporal cell signaling changes that occur in the normal—> dysplasia—> early carcinoma continuum of oral oncogenesis. For example, RNA seq showed increased transcription of VEGFa, a quantitative finding, but ST was able to show spatio‐temporal expansion of this increased signaling. First, elevated expression was localized to the upper layer of the epithelium in the transition from normal to dysplasia, which is thought to promote additional cellular proliferation of the basal layer. Next, as dysplasia progresses to cancer, the signaling switches to the basal layer, thought to be at the direction of and indicative of epithelial‐stromal cross talk thought to occur late in oncogenesis, which helps to facilitate invasion [26]. The significance of this finding is that spatial cell: cell signaling changes predispose and precede development of cancer, and ST is one such tool that can identify these hallmarks of disease progression on a molecular level. In addition, the spatial context from ST has afforded insight into the different spatial and molecular differences of oral dysplasia based upon their outcomes [39]. ST studies have shown transcriptionally distinct domains within lesions based upon cell and transcriptional activity, such as proliferative, fibrotic, immune “hot” or “cold” niches that all exist in different aspects of the lesions contributing to the proliferation of aberrant cells and epithelial‐stromal crosstalk and invasion. These niches also can be distinguished on a transcriptional basis from OPMD to periodontal or other inflammatory conditions, showing value and proof of concept for inclusion of ST to predict oral cancer from OPMD [40]. Thus, ST represents the ideal diagnostic tool to provide a spatio‐temporal profile of OPMDs to round out and complete the framework according to principles set forth by the NCI HTAN's Precancer Genome Atlas Program.

## 2 Discussion: Toward an Artificial Intelligence Model for OPMD Progression Prediction

We propose thinking of OPMD malignant transformation prediction as a multimodal machine learning problem organized around two complementary goals. In the first goal, a learned image‐molecular features association would be developed through years of matching histologic images to molecular sequencing data. This learned image: molecular association would also be anchored to patient outcomes. Once a robust histologic image‐molecular data association is developed and matched to outcomes, the second goal would be to distill that representation into a deployable test that can be inferred from routine clinical biopsy and patient covariates. To achieve both goals, we propose a machine learning “Teacher‐Student” model. The Teacher would learn to associate certain patterns of staining intensity, morphology, cell structure, and architecture and microenvironment changes such as those that occur across the epithelial and stromal compartments, and how they pair with results of multiomic complex sequencing. For example, while genomic, epigenomic, and transcriptional changes themselves may not be visualized, their phenotypic representations may be evident in architectural, maturational, or nuclear changes in the cell or cell layers, architectural changes in the basal cell‐stromal interface such as invasion, rete‐ridge formation, loss of basal cell polarity, cytologic atypia, immune cell infiltration, and keratinization patterns that can be linked to molecular results. As the Teacher learns these associations and distills the complex data and images to a simpler pairing of features and molecular states, this could be then taught to a Student model that could be deployed for clinical use. The intentional design of this framework and planned machine learning integration should create a model that creates “learned molecular‐image associations” that is anchored to patient outcomes (regression, stability, progression to high‐grade requiring resection or progression to malignancy) and over what time this occurs.

The second step is a Student model designed for clinical deployment. The Student is trained to predict the Teacher's molecular risk embedding, or numerical representation generated from molecular assays to the paired H&E whole‐slide image and key patient covariates (which can leverage EHR data). Conceptually, this teaches the model to infer underlying molecular state from morphology and microenvironmental context, features that are already assessed qualitatively by pathologists, while leveraging multiomics supervision to make that inference quantitative and progression relevant. Once trained, the Student no longer requires molecular assays at inference time: it can accept a standard diagnostic H&E slide and patient specific covariates and output a calibrated risk estimate, ideally expressed as a time‐to‐event prediction or risk curve over time (Figure 2). This design directly addresses the central translation barrier in OPMD prognostics: molecular assays are powerful but costly and logistically variable, whereas H&E is universally available, inexpensive, and already embedded in clinical workflows. By using multiomics to train the model but not to run the model, this framework aims to integrate the necessary molecular backbone with the already practical clinical biopsy workflow, enabling a computational prognostic test without additional staining or specialized laboratory infrastructure.

**FIGURE 2:**
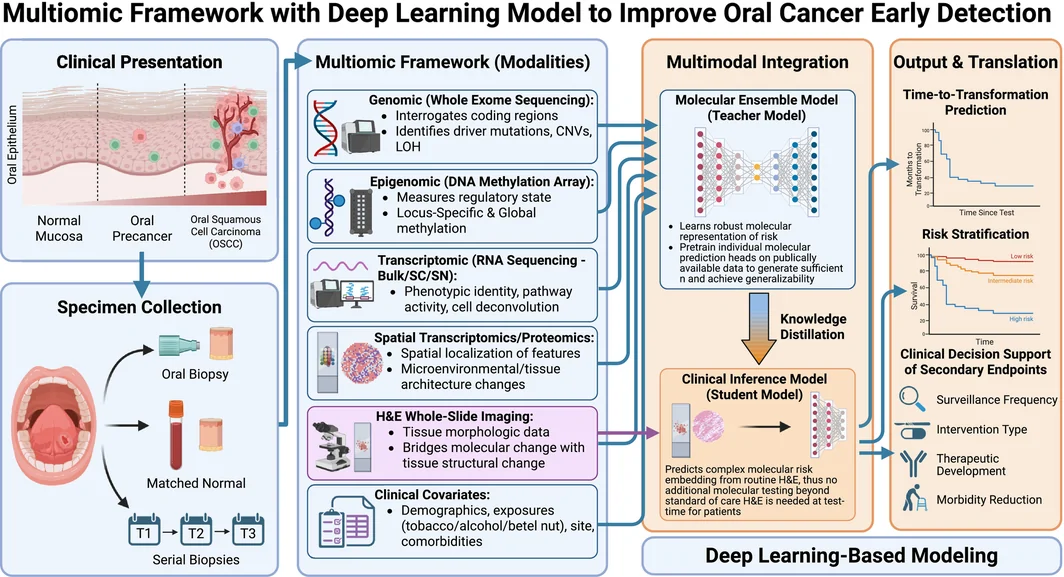
An integrated, multimodal framework combining patient associated and specimen derived data with multimodal deep learning matching features to outcomes to better predict oral cancer risk. [Color figure can be viewed at wileyonlinelibrary.com]

This machine learning integration is ambitious and will likely take 6–8 years of multi‐institutional study. Pretraining on publicly available datasets can improve modeling and ensure that model development occurs such that integration is performed in a way that preserves the relationship between the test and the outcome measured. This should provide deep learning outcomes that can achieve both performance and biological interpretability. In practice, this means avoiding unsupervised analysis without pretraining and instead incorporating biologically grounded preprocessing and structured training regimes that encourage the model to learn stable signal rather than technical artifacts.

For example, rather than training a methylation‐based unsupervised neural network to reconstruct all ~930 000 CpG values, one can incorporate features that reflect biologically meaningful organization—such as differentially methylated regions or global hypomethylation burden in specific predetermined loci. In addition, pretraining on large public methylation datasets with supervised objectives, like tissue or cancer subtype discrimination, or the algorithm learns separable, biologically meaningful inputs to coarsely define the model limits before fine‐tuning on OPMD cohorts.

Many challenges exist to see such a proposed framework to clinical utilization. We expect that there will be heterogeneity in the molecular alterations that cause OPMD progression and that many distinct subtypes of “progressor” molecular profiles will be found. As a result of our expectation of overlapping image‐molecular association, we include patient covariates and serial biopsies over time to improve patient specific prediction when image‐molecular associations overlap or are equivocal. We believe that integration of patient covariates will help a “Student” model to improve patient prediction and risk stratification over histology alone. In addition, OPMD cohorts represent a classic *p* ≫ *n* setting, where predictor variables (*p*) outweigh the number of samples (*n*). In this context, each patient generates far more molecular measurements than any feasible clinical trial can enroll in samples. Without careful constraints, a model may overfit cohort‐specific idiosyncrasies, such as batch effects, site‐specific protocols, demographic imbalances, or sample handling differences, leading to poor generalization when applied to new sites or new patient populations. This can be overcome with intention in study design. First, dimensionality reduction is essential, but it must be biologically meaningful: each modality should be compressed into more simplified representations, or numerical embeddings, that preserve progression‐relevant information while reducing sensitivity to noise. Second, regularization and robust validation strategies must be incorporated from the outset, including site‐aware splits, batch‐aware modeling, and prospective or external validation whenever possible.

There are many other limitations of this manuscript and proposed framework. Multiomic sequencing is costly, complex, and time intensive. Such a framework proposed here would require multiple university cores or proprietary companies to carry out the different types of sequencing should all presented assays be utilized. Whole exome, RNA Sequencing, and Spatial transcriptomics can be completed on FFPE specimens, but methylation analyses require fresh, frozen tissue, which complicates tissue processing and protocols. In addition, spatial transcriptomics is costly and not yet as feasible to complete as exome or RNA sequencing. Successful implementation requires a robust and complex standard operating protocol of tissue and data handling. Data must be collected over time with serial biopsy, and this must be carried out long enough to detect enough transformation events (transformation to high grade requiring intervention or to malignancy) for robust model development.

Additionally, to obtain a large enough sample size, multi‐institutional collaboration is paramount and would require high‐level national grants, significant standardization in patient accrual, sampling, tissue preservation, sequencing, data analysis and storage. Next, consideration should be made as to whether the tissue analysis is done at one central location or across institutions. Examination across sites could help to mitigate potential batch effects from the use of one institution or companies' resources and could help to survive external validation and improve its clinical utility.

If this all comes to fruition, the next great barrier is to see whether common molecular profiles for progressing versus nonprogressing lesions can be determined. More importantly, this risk stratification would need to improve on the current best negative predictive value, which is about 87% [22]. Finally, and probably the greatest limitation of this and all current proprietary investigational tools is the challenge of providing a cost effective, efficacious point of care test that can be commercialized. Even if we can improve risk stratification using complex sequencing, we may not be able to predict this molecular output from histology. Not all molecular changes produce morphologic changes. Therefore, although molecular assays may improve stratification, if they don't manifest these changes, this is a significant limitation to inferring these complex features to histology. This is the largest hurdle to the development of any tool, algorithm, or device and remains the greatest impediment to providing a widely available, cost effective, simple tool to predict malignant transformation of oral precursor lesions.

In conclusion, this framework provides an HTAN aligned method of tissue analysis and data handling to optimize outputs in analyzing molecular events of lesions as they progress to cancer. It mirrors industry standards of trying to infer complex sequencing back to h&E such as Bioptimus and Noetik, leverages machine learning, provides a framework that could be reproducible across sites for the potential to maximize the opportunity to develop a prediction tool to pass external validation and meet the unmet need in oral cancer prediction.

## Funding

The authors have nothing to report.

## Ethics Statement

This article is a review of NCI principles and publications utilizing these principles. No human subjects were studied as part of this manuscript and therefore no IRB approval is required.

## Conflicts of Interest

The authors declare no conflicts of interest.

## Data Availability

This manuscript presents a conceptual framework; no data were generated in the process. All investigation related to the manuscript is present in the presented text, table, or figures.
